# Publisher Correction: Mechanisms of inhibition and activation of extrasynaptic αβ GABA_A_ receptors

**DOI:** 10.1038/s41586-022-04663-8

**Published:** 2022-03-24

**Authors:** Vikram Babu Kasaragod, Martin Mortensen, Steven W. Hardwick, Ayla A. Wahid, Valentina Dorovykh, Dimitri Y. Chirgadze, Trevor G. Smart, Paul S. Miller

**Affiliations:** 1 Department of Pharmacology, University of Cambridge, Cambridge, UK; 2 MRC Laboratory of Molecular Biology, Cambridge, UK; 3 Department of Neuroscience, Physiology and Pharmacology, University College London, London, UK; 4 Cryo-EM Facility, Department of Biochemistry, University of Cambridge, Cambridge, UK

**Keywords:** Ion channels in the nervous system, Molecular neuroscience, Cryoelectron microscopy

Correction to: *Nature* 10.1038/s41586-022-04402-z Published online 9 February 2022

In the version of this article initially published, the first sentence of the Author contributions section was truncated. The section has been amended to begin “V.B.K. performed protein purification, atomic model building and structural interpretation.” The error has been corrected in the HTML and PDF versions of the article.

