# Supplementary material for: Mechanisms of inhibition and activation of extrasynaptic αβ GABAA receptors
Source: Nature. 2022 Feb 9;602(7897):529–33. doi: 10.1038/s41586-022-04402-z (PMC8850191; doi:10.1038/s41586-022-04402-z)
Supplement: Supplementary file 1 — Reporting Summary [file 41586_2022_4402_MOESM1_ESM.pdf]

nature research

Corresponding author(s): Paul S. Miller
Trevor G. Smart

Last updated by author(s): Jun 7, 2021

Reporting Summary

Nature Research wishes to improve the reproducibility of the work that we publish. This form provides structure for consistency and transparency in reporting. For further information on Nature Research policies, see our Editorial Policies and the Editorial Policy Checklist.

Statistics

For all statistical analyses, confirm that the following items are present in the figure legend, table legend, main text, or Methods section.

n/a	Confirmed	
☐	☒	The exact sample size (*n*) for each experimental group/condition, given as a discrete number and unit of measurement
☐	☒	A statement on whether measurements were taken from distinct samples or whether the same sample was measured repeatedly
☐	☒	The statistical test(s) used AND whether they are one- or two-sided<br>*Only common tests should be described solely by name; describe more complex techniques in the Methods section.*
☒	☐	A description of all covariates tested
☒	☐	A description of any assumptions or corrections, such as tests of normality and adjustment for multiple comparisons
☐	☒	A full description of the statistical parameters including central tendency (e.g. means) or other basic estimates (e.g. regression coefficient) AND variation (e.g. standard deviation) or associated estimates of uncertainty (e.g. confidence intervals)
☐	☒	For null hypothesis testing, the test statistic (e.g. *F*, *t*, *r*) with confidence intervals, effect sizes, degrees of freedom and *P* value noted<br>*Give P values as exact values whenever suitable.*
☒	☐	For Bayesian analysis, information on the choice of priors and Markov chain Monte Carlo settings
☒	☐	For hierarchical and complex designs, identification of the appropriate level for tests and full reporting of outcomes
☒	☐	Estimates of effect sizes (e.g. Cohen's *d*, Pearson's *r*), indicating how they were calculated

Our web collection on statistics for biologists contains articles on many of the points above.

Software and code

Policy information about availability of computer code

Data collection	Clampex 10.2, FEI EPU
Data analysis	Warp 1.09, cryoSPARC v3.2.0, UCSF Chimera v1.13.1, Pymol v2.1.1, Coot 0.9.3, Phenix 1.19.1, MolProbity, HOLE, Clampfit 10.2, Graphpad Prism 9, Origin 6, WinEDR

For manuscripts utilizing custom algorithms or software that are central to the research but not yet described in published literature, software must be made available to editors and reviewers. We strongly encourage code deposition in a community repository (e.g. GitHub). See the Nature Research guidelines for submitting code & software for further information.

Data

Policy information about availability of data

All manuscripts must include a data availability statement. This statement should provide the following information, where applicable:

- Accession codes, unique identifiers, or web links for publicly available datasets
- A list of figures that have associated raw data
- A description of any restrictions on data availability

Atomic model coordinates for alpha-CBTx/Zn2+, GABA/Zn2+ and GABA-bound structures have been deposited in the Protein Data Bank with accession codes 7PC0, 7PBZ, 7PBD, respectively. Cryo-EM density maps have been deposited in the Electron Microscopy Data Bank with accession codes EMD-13315, EMD-13314, EMD-13290 respectively.

Field-specific reporting

Please select the one below that is the best fit for your research. If you are not sure, read the appropriate sections before making your selection.

☒ Life sciences ☐ Behavioural & social sciences ☐ Ecological, evolutionary & environmental sciences

For a reference copy of the document with all sections, see nature.com/documents/nr-reporting-summary-flat.pdf

Life sciences study design

All studies must disclose on these points even when the disclosure is negative.

Sample size	No statistical methods were used to estimate appropriate sample size.
Data exclusions	Following standard cryoSPARC processing pathways, best representative 2D and 3D classes were selected for the final particle reconstructions. Poor quality and irrelevant other classes were discarded.
Replication	Attempts to replicate/reproduce the data were successful as detailed in the electrophysiology replicates. For cryo-EM Two independent maps of each cryo-EM sample were generated in order to estimate resolution according to the recommended procedures in the field (the 'gold standard').
Randomization	EM particle sets were randomly split for the purposes of estimating overall resolution. Otherwise randomization was not relevant to these studies.
Blinding	Blinding was not relevant to this study.

Reporting for specific materials, systems and methods

We require information from authors about some types of materials, experimental systems and methods used in many studies. Here, indicate whether each material, system or method listed is relevant to your study. If you are not sure if a list item applies to your research, read the appropriate section before selecting a response.

Materials & experimental systems

n/a	Involved in the study	
☐	☒	Antibodies
☐	☒	Eukaryotic cell lines
☒	☐	Palaeontology and archaeology
☒	☐	Animals and other organisms
☒	☐	Human research participants
☒	☐	Clinical data
☒	☐	Dual use research of concern

Methods

n/a	Involved in the study	
☒	☐	ChIP-seq
☒	☐	Flow cytometry
☒	☐	MRI-based neuroimaging

Antibodies

Antibodies used	Rho-1D4 antibody was purchased from the University of British Columbia. The megabody Mb25 was obtained from the laboratory of Professor Jan Steyeart (VUB). Nanobody Nb25 was made in the lab.
Validation	Rho-1D4 validated by ability to purify GABA-A-R. The nanobody Nb25, used to design the megabody Mb25 as described in methods, was characterised and published elsewhere (PMID: 28991263). Mb25 has been characterised and published elsewhere (PMID: 33408403).

Eukaryotic cell lines

Policy information about cell lines

Cell line source(s)	HEK 293S GnTI- cells were obtained from ATCC.
Authentication	Further authentication was not performed for this study.
Mycoplasma contamination	Mycoplasma testing was not performed for this study.
Commonly misidentified lines (See ICLAC register)	No commonly misidentified cell lines were used in this study.
